# DNA Barcoding and geographical scale effect: The problems of undersampling genetic diversity hotspots

**DOI:** 10.1002/ece3.6733

**Published:** 2020-09-01

**Authors:** Álvaro Gaytán, Johannes Bergsten, Tara Canelo, Carlos Pérez‐Izquierdo, Maria Santoro, Raul Bonal

**Affiliations:** ^1^ Department of Ecology Environment and Plant Sciences Stockholm University Stockholm Sweden; ^2^ Research Group on Genetic and Cultural Biodiversity – IREC – (CSIC, UCLM, JCCM) Ciudad Real Spain; ^3^ Department of Zoology Swedish Museum of Natural History Stockholm Sweden; ^4^ Forest Research Group INDEHESA University of Extremadura Plasencia Spain

**Keywords:** cytochrome c oxidase subunit I, glacial refugia, intraspecific genetic divergence, Lepidoptera

## Abstract

DNA barcoding identification needs a good characterization of intraspecific genetic divergence to establish the limits between species. Yet, the number of barcodes per species is many times low and geographically restricted. A poor coverage of the species distribution range may hamper identification, especially when undersampled areas host genetically distinct lineages. If so, the genetic distance between some query sequences and reference barcodes may exceed the maximum intraspecific threshold for unequivocal species assignation. Taking a group of *Quercus* herbivores (moths) in Europe as model system, we found that the number of DNA barcodes from southern Europe is proportionally very low in the Barcoding of Life Data Systems. This geographical bias complicates the identification of southern query sequences, due to their high intraspecific genetic distance with respect to barcodes from higher latitudes. Pairwise intraspecific genetic divergence increased along with spatial distance, but was higher when at least one of the sampling sites was in southern Europe. Accordingly, GMYC (General Mixed Yule Coalescent) single‐threshold model retrieved clusters constituted exclusively by Iberian haplotypes, some of which could correspond to cryptic species. The number of putative species retrieved was more reliable than that of multiple‐threshold GMYC but very similar to results from ABGD and jMOTU. Our results support GMYC as a key resource for species delimitation within poorly inventoried biogeographic regions in Europe, where historical factors (e.g., glaciations) have promoted genetic diversity and singularity. Future European DNA barcoding initiatives should be preferentially performed along latitudinal gradients, with special focus on southern peninsulas.

## Graphical abstract

A poor coverage of the species distribution range may hamper identification, and the genetic distance between some query sequences and reference barcodes may exceed the maximum intraspecific threshold for unequivocal species assignation. Our results support GMYC as a key resource for species delimitation within poorly inventoried biogeographic regions in Europe, where historical factors (e.g., glaciations) have promoted genetic diversity and singularity. Future European DNA barcoding initiatives should be preferentially performed along latitudinal gradients, with special focus on southern peninsulas.

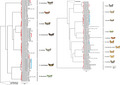

## 1 INTRODUCTION

A decade and a half ago, DNA barcoding was presented as a novel system to provide wide‐scale and quick species identification using certain gene sequences as molecular species‐specific tags (Hebert, Cywinska, Ball, & de Waard, 2003). Since then, the number of species sequenced has increased exponentially, and DNA barcodes are available for almost 200K named species in international databases such as the Barcode of Life Data Systems BOLD (Ratnasingham & Hebert, 2007). DNA barcoding often allows for the identification of morphologically cryptic species and individuals at life stages difficult to determine morphologically (e.g., insect larvae) (Ahrens, Fabrizi, Šípek, & Lago, 2013; Bonal, Muñoz, & Vogler, 2011). It has boosted biodiversity inventories and environmental monitoring and constitutes a useful tool in taxonomy, ecology, agriculture, and conservation as well as for customs, police, food, and feed control (Bergsten et al., 2012; Jinbo, Kato, & Ito., 2011; Savolainen, Cowan, Volger, Roderick, & Lane, 2005). The Barcoding of Life initiative constitutes a historical feat, but this success does not mean that the method is free of shortcomings (Bergsten et al., 2012; Berthier, Chapuis, Moosavi, Tohidi‐Esfahani, & Sword, 2011; Dubey, Michaux, Brünner, Hutterer, & Vogel, 2009; Nicholls, Challis, Mutun, & Stone, 2012). One of them is the potential decline of identification accuracy as intraspecific genetic divergence increases along with the geographical scale (Meyer & Paulay, 2005; Bergsten et al., 2012, but see Lukhtanov, Sourakov, Zakharov, & Herbert, 2009). In this study, we approach whether this problem aggravates when genetic diversity hot spots are undersampled.

In animals, a 648‐bp section of the universal mitochondrial gene encoding for the protein cytochrome c oxidase subunit I (COI) has been adopted as the standard barcode (Hebert et al., 2003; Ratnasingham & Hebert, 2007). The logic behind DNA barcoding relies on the structure of genetic variability above and below the species level. Individuals of the same species display lower levels of genetic divergence among themselves compared with heterospecific individuals (Hajibabaei, Singer, Hebert, & Hickey, 2007; Hebert et al., 2003). Any genetic threshold used for identification of queries is arbitrary, ideally optimized for the dataset in question, and may for instance be 1%, 2%, or 3% (Collins & Cruickshank, 2012; Lemos, Fulthorpe, Triplett, & Roesch, 2011; Ratnasingham & Hebert, 2007). BOLD identification engine for instance uses 1% for species‐level taxon assignment (Ratnasingham & Hebert, 2007). However, a plethora of methods have been proposed for identification of unknowns against a reference library, tree‐based, distance‐based, character‐based, but few improve noticeably on a standard “best close match” sequence distance strategy (Spouge, 2016). Many of the more sophisticated methods are also too slow to be applicable to the growing needs of taxon assignment from the DNA metabarcoding community.

While the presence of pseudogenes (Berthier et al., 2011; Dubey et al., 2009), former hybridization, or incomplete lineage sorting (Nicholls et al., 2012) may mislead identification, one of the main caveats of DNA barcoding is not related with the evolutionary history of the genes, but with the geographical distribution of the samples. When the geographical scale increases, intraspecific divergence increases, and the distance to the closest related taxa decreases, which results in more ambiguous specimen identification (Bergsten et al., 2012).

The geographical scale effect on intraspecific divergence is based on the well‐known concept of genetic isolation by distance (Wright, 1943), but the relationship between genetic divergence and distance may differ geographically. Taking Europe as an example, studies with different types of organisms have demonstrated that, far from being homogeneously distributed, genetic diversity is concentrated in certain areas of the continent (Avise, 2000; Hewitt, 1996; Schmitt, 2007). Thus, for a given spatial distance between the sampling sites of two DNA barcodes, the genetic distance could be higher if at least one of them comes from a genetic diversity hot spot.

In Europe, apart from taxon‐specific projects, large national barcoding initiatives are all in north and central Europe, for example, Germany (Gemeinholzer et al., 2011), the Netherlands (Beentjes, Speksnijder, Van der Hoorn, & Van Tol, 2015), Norway (Ekrem et al., 2015), and Finland (Huemer, Mutanen, Sefc, & Hebert, 2014; Pentinsaari, Hebert, & Mutanen, 2014), far from the southern Peninsulas (Iberia, Italy, and the Balkans) that host higher levels of biodiversity, endemism, and genetic diversity (Geiger et al., 2014; Hewitt, 1996; Murienne & Giribet, 2009; Pinto, Muñoz, Chávez‐Galarza, & De la Rúa, 2012). In fact, when a few smaller‐scale barcoding initiatives have been carried out in southern Europe for specific groups (like butterflies in the Iberian Peninsula of freshwater fish around the Mediterranean Basin), the results have revealed a high genetic richness and distinctiveness and the existence of a number of potential cryptic species (Dincă et al., 2015; Geiger et al., 2014).

In this study, we analyzed the geographical scale effect on intraspecific genetic distance using as study model a group of Heteroceran Lepidoptera (i.e., moths) whose caterpillars feed on oak (*Quercus* spp.) leaves. These moth species are widely distributed over most parts of Europe (Camus, 1936–1954). We could thus download a high number of DNA barcodes from the public repository BOLD that were pooled in the analyses with newly sequenced Iberian samples. Previous reports on Lepidoptera have shown little intraspecific divergence at a large geographical scale between Central and Northern Europe (Huemer et al., 2014). In this study, we included individuals from the south of the continent to assess the effect of genetic diversity hot spots on intraspecific genetic distance and identification success. Our concrete objectives were as follows:

1. To analyze to which extent the availability of DNA barcodes is biased toward central and northern Europe.
2. To know whether, in pairwise sequence comparisons, for any given spatial distance, the genetic divergence is higher if at least one of the sequences comes from a southern European peninsula.
3. To reconstruct a COI gene tree to assess the geographical distribution of genetic diversity on the continent and the presence of monophyletic clades (intraspecific distinct lineages and potential cryptic species) exclusive of southern Europe.

## 2 METHODS

### 2.1 Study system and field sampling

**Figure 1:**
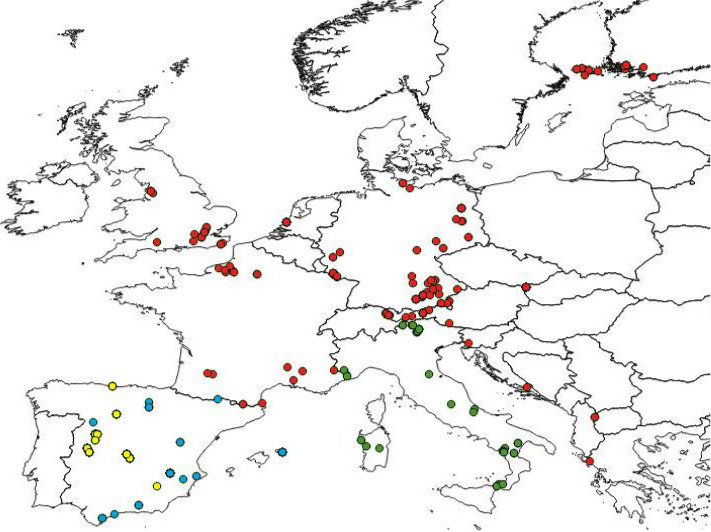
Geographical distribution of sampled localities including BOLD records (Europe: red; Iberian Peninsula: blue; and Italy: green) and new localities sampled for this study (yellow)

The Heteroceran Lepidoptera (moths) whose caterpillars feed on *Quercus* spp. leaves were used as study model. There is a high diversity of lepidopterans linked to the *Quercus* genus as host plant. The most abundant oak herbivore families are noctuids, tortricids, erebids, geometrids, and nolids, which start feeding on the new shoots in early April (Elkinton et al., 1996). The community of caterpillars also changes during the season, with tortricids the first to feed on the new shoots and geometrid species the last (Soria, 1988). Herbivory damage on oak differs among years too, being able to have outbreaks under specific conditions of temperature and climate (Schroeder & Degen, 2008). These insects are widely spread over Europe and have been recorded feeding on different oak species (Führer, 1998; Soria, 1988). The number of DNA barcodes available for the southern European peninsulas (Balkans, Italy, and Iberia) at public repositories was very low compared with the rest of continental Europe (see below). Thus, we carried out a field campaign in Spain to reduce such an imbalance. We sampled 10 oak forests along a latitudinal gradient (Figure 1) from April to June 2017, the period of maximum activity of oak defoliating caterpillars in the Iberian Peninsula (Soria, 1988). Oak branches were shaken and the falling caterpillars collected on a white cloth of a fixed surface placed beneath (see Ruiz‐Carbayo, Bonal, Espelta, Hernández, & Pino, 2017 for a detailed description of the sampling methodology). The caterpillars collected at each oak were placed within a plastic box and taken to the laboratory, where they were housed individually in Petri dishes and fed with fresh oak leaves. Caterpillars were identified to species level based on morphological characters following guides and dichotomous keys (Gaytán, Canelo, González‐Bornay, Pérez‐Izquierdo, & Bonal, 2018; Gomez de Aizupura, 2002). In those few cases in which the caterpillar could not be identified, it was raised to the adult stage and then identified using guides (Fibiger, 1997; Goater, Ronkay, & Fibiger, 2003; Sihvonen & Skou, 2015). In total, 21 species of 9 families were collected (Table 1). All specimens (caterpillars and adults) were stored in 1.5‐ml Eppendorf tubes filled with 96% alcohol for further molecular analyses.

**Table 1:** Maximum intraspecific genetic divergence (K2P%) for each species in the pairwise comparisons between populations of Europe (EUEU), Europe–Iberia (EUIB), Europe–Italy (EUIT), and Iberia (IBIB)

FAMILY	SPECIES	EUEU	EUIB	EUIT	IBIB
Tortricidae	*Archips xylosteana*	1.4	1.8	1.1	0.7
Nolidae	*Bena bicolorana*	0.4	1.0	NA	1.0
Erebidae	*Catocala nymphagoga*	NA	2.2	0.4	2.0
Geometridae	*Colotois pennaria*	0.0	2.8	NA	0.6
Geometridae	*Colotois pennaria carbonii*	0.7	NA	0.7	NA
Noctuidae	*Dryobotodes eremita*	0.3	0.8	0.3	1.1
Noctuidae	*Dryobotodes monochroma*	0.5	0.8	NA	1.1
Geometridae	*Ennomos quercaria*	NA	NA	NA	1.8
Geometridae	*Eupithecia cocciferata*	NA	NA	NA	0.5
Erebidae	*Euproctis chrysorrhoea*	2.3	2.3	2.3	2.2
Lasiocampidae	*Malacosoma neustria*	0.5	0.9	0.5	0.7
Nolidae	*Nycteola columbana*	NA	0.7	0.2	1.0
Noctuidae	*Orthosia cruda*	1.2	1.0	1.2	0.0
Geometridae	*Peribatodes ilicaria*	2.7	3.4	NA	3.9
Tortricidae	*Tortricodes alternella*	0.2	2.0	0.2	1.1
Tortricidae	*Tortrix viridana*	0.4	0.9	0.7	0.9
Noctuidae	*Watsonalla uncinula*	NA	1.9	0.2	0.9
Noctuidae	*Xanthia ruticilla*	0.6	0.9	1.5	0.6

Abbreviation: NA = not applicable.

### 2.2 Molecular laboratory work

DNA of 384 individuals of different species and Iberian localities was extracted using commercial extraction kit (EZNA^®^ Tissue DNA Kit) according to manufacturer's instructions. For all of them, a fragment of the mitochondrial gene COI was amplified using the universal primer pair LCO1490/HCO2198, common in DNA barcoding (see Folmer, Black, Hoeh, Lutz, & Vrijenhoek, 1994 for details on the primer sequences and PCR protocols). Sequencing was performed using Big‐Dye (Perkin‐Elmer) technology and an ABI3700 sequencer. We obtained good quality DNA sequences for 375 individuals (21 species); the DNA from the remaining 9 individuals could not be further used due to sequencing failures. Sequence chromatograms were assembled, inspected individually, and edited using Sequencher 4.6 (Gene Codes Corp.). The final alignment length after trimming was 658 bp. To rule out the presence of nuclear mitochondrial insertions (numts), we translated all the sequences into amino acids using the software MacClade (Maddison & Maddison, 2005). After translation, we did not find any stop codons indicating the presence of numts (no stop codons can be present in the intronless mitochondrial COI gene). Moreover, there were additional evidences against the presence of numts. In no case, the sequences of the same species were paraphyletic and intraspecific genetic divergence was lower than that expected if some of the sequences were numts (Bonal et al., 2018).

### 2.3 Preparation of databases of DNA barcodes

Sequences of the 375 individuals collected during our field sampling were queried in BOLD search engine (Ratnasingham & Hebert, 2007) to double‐check the species identity in those specimens previously determined according to morphology. We used the option All Barcode Records in BOLD, as all our sequences were longer than 500 bp and doing so the query sequences are compared with a larger number of reference barcodes. Besides, we mined BOLD searching for COI DNA barcodes of the same species that we had sequenced in the Iberian Peninsula; in total, we could download 277 public sequences (available in January 2018), which were used in further analyses (see Appendix S1 for BOLD process IDs).

The final database pooling the sequences downloaded from BOLD with the new Iberian sequences added up to 652 COI barcodes: 21 taxa from 136 localities in 14 European countries (Figure 1). For the data downloaded from BOLD, we considered two sequences to come from the same locality when the name for that field site was identical in the database or when the coordinates showed that the specimens had been collected within a distance lower than 5 km. We chose this threshold because, given that these species of oak‐feeding moths are not migratory and have limited dispersal abilities (Ruiz‐Carbayo et al., 2017), individuals within this range may belong to the same population.

### 2.4 Calculation of intraspecific genetic distance and geographical distance among populations

For each species, the maximum intraspecific genetic divergence and the geographical distance between localities were needed for further analyses (see below the section on statistical tests). For measuring genetic divergence, we first aligned all the sequences of each species separately using MUSCLE software (Edgard, 2004) as implemented in MEGA 7 (Kumar, Strecher, & Tamura, 2016; default values). For each separate species alignment, all pairwise genetic distances were calculated using the Kimura 2‐parameter model (K2P%; Kimura, 1980) as implemented in MEGA 7 (Kumar et al., 2016); we used K2P% because this is the method more frequently used in DNA barcoding studies (Bergsten et al., 2012; Gunay, Alten, Simsek, Aldemir, & Linton, 2015; Shen, Guan, Wang, & Gan, 2016). The maximum genetic divergence for each pair of populations was the maximum genetic distance (K2P%) between any pair of individuals of those two populations. Pairwise geographical distances between populations were calculated using QGIS 2.18.9 (QGIS Development Team, 2009) and later corroborated using the cosine‐haversine formula (Robusto, 1957).

### 2.5 Molecular single‐gene species delimitation

The sequences of each species alignment were collapsed into unique haplotypes using the online.fasta sequence toolbox FaBox (Villesen, 2007). We discarded four species either because no sequences were available from any of the southern European peninsulas (*Orthosia cerasi*) or elsewhere on the continent (*Eupithecia massiliata*, *Phycita torrenti*, and *Dryobota labecula*). We removed them because those missing data precluded any pairwise comparison between populations from at least one southern Peninsula and the rest of the continent. Our final database included a total of 18 taxa, 17 species plus the subspecies *Colotois pennaria* ssp. *carbonii*, which was treated as distinct species in the analyses on intraspecific genetic divergence (Table 1). We did so to be conservative and avoid inflating intraspecific genetic divergence in *C. pennaria*. The ssp. *carbonii* is morphologically distinct (Sihvonen & Skou, 2015), and so the intraspecific divergence would be expected to be higher than in those species in which no subspecies have been described so far. The alignments of the unique haplotypes of each of the 18 species were visually inspected one by one in MEGA 7 (Kumar et al., 2016); we corroborated that there had been no errors and all sequences differed in at least one base. Then, the haplotypes of all species were pooled together and subsequently aligned again; this was the basic and final alignment used for species delimitation with the Generalized Mixed Yule Coalescent (GMYC) method.

The GMYC single‐locus method identifies the number of independent operational taxonomic units (OTUs) or putative species present in a sample of sequences under the maximum likelihood solution (Fujisawa & Barraclough, 2013; Pons et al., 2006). Some OTUs can correspond to cryptic species or isolated genetic lineages; thus, this sort of analyses may well illustrate the patterns of geographical genetic variability of these insects in Europe. GMYC requires an ultrametric gene tree as input, which was built using the software Bayesian Evolutionary Analysis Sampling Trees (BEAST 1.7.5; Drummond, Suchard, Xie, & Rambaut, 2012). To select appropriate partitioning scheme and substitution model for the three‐codon positions of COI, we used PartitionFinder version 1.1.1 (Lanfear, Calcott, Ho, & Guindon, 2012). We tested between the available models in BEAST using the Bayesian information criterion (BIC) and between all possible partitioning schemes. PartitionFinder supported three separate partitions for the codon positions, and three different models were selected according to the BIC scores (equal‐frequency Tamura–Nei model with Gamma correction (G), Hasegawa Kishino Yano model with invariable sites (I), and equal‐frequency Tamura–Nei model with Gamma correction (G) for 1st, 2nd, and 3rd codon positions, respectively). We used a strict clock model with rate fixed to 1 and a constant size coalescent tree prior as this could be considered conservative toward the null model when testing against the GMYC model in a likelihood ratio test (Monaghan et al., 2009). The effects of tree reconstruction method and model for the GMYC results have been investigated and, in general, a Bayesian estimation under a coalescent tree prior has performed well in comparisons (Monaghan et al., 2009; Talavera, Dincă, & Vila, 2013; Tang, Obertegger, Fontaneto, & Barraclough, 2014). Talavera et al. (2013) found no difference in GMYC results between using a strict or relaxed clock model for inferring the input ultrametric tree. We ran two Markov chain Monte Carlo (MCMC) runs each with 10 million generations, sampled every 1,000 generations, which were merged using LogCombiner. Convergence and effective sample size (ESS) values for sampled model parameters were monitored in Tracer version 1.6 (Rambaut, Suchard, Xie, & Drummond, 2014). TreeAnnotator was used to select the maximum clade credibility tree (MCC tree) from the sampled trees (burn‐in = 0.25) with posterior median values used for node heights.

**Figure 2:**
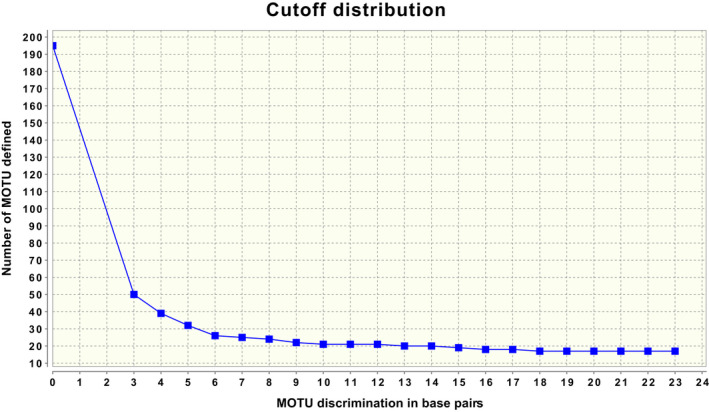
Number of molecular operational taxonomic units (MOTU) delimited by jMOTU 4.1 over a range of cutoff values in unit of base pair differences. jMOTU is a distance‐based method that calculates pairwise distances between sequences and then clusters the sequences into MOTUs using single‐linkage clustering for each user‐supplied cutoff value (Jones et al., 2011). Resulting clusters are not affected by input sequence order and no members of a given OTU will be closer than the cutoff to any member of any other cluster. The Java program is speed‐optimized by a preclustering step of exact subsequences and can therefore (in contrast to ABGD) be run on a reduced haplotype‐collapsed dataset without any effect on the final clusters. jMOTU does not use a substitution model that corrects for homoplastic substitutions but calculates only exact base pair differences. We ran the jMOTU analysis with the haplotype‐collapsed dataset (cropped to 624 bp and filtered for sequences >500 bp long due to the sensitivity of jMOTU to sequence length variation in the dataset (see Stahlhut et al., 2013, https://doi.org/10.1186/1472‐6785‐13‐2), which we also noted), a low BLAST identity filter of 95%, and a percentage of minimum sequence length overlap of 90% following the software manual's recommendations. We calculated how the number of delimited MOTUs varied over a cutoff range between 3 and 23 bp, which for 624 bp equals to 0.5%–3.7% in genetic divergence. At all cutoff values above 18 bp (>2.9%), jMOTU delimits 17 clusters corresponding to named species, similar to ABGD at prior maximum intraspecific divergence values from 0.03 and above (See Table 2). At decreasing cutoff values from 17 to 6 bp (2.7%–1.0%), there is a gradual increase in the number of delimited clusters from 18 to 26 MOTUs. The transition mirrors well the recursive partitioning results from ABGD from 19 to 25 clusters in prior space between 0.023 and 0.005 (See Table 2). The 25 clusters delimited by jMOTU at a 7 bp (1.1%) cutoff are identical to the 25 units delimited by single‐threshold GMYC and differ from the 25 groups delimited by ABGD at a prior of 0.005 only by the same two exceptions as between GMYC and ABGD: *Ennomos quercaria* is subdivided into two and not three groups and instead *Tortricodes alternella* is subdivided into two groups

The maximum clade credibility (MCC) tree with branch lengths was imported in R version 3.3.1 (R Core Team, 2016), and the GMYC analysis was conducted using the splits package version 1.0 (Ezard, Fujisawa, & Barraclough, 2009), assisted by the ‘APE’ package (Paradis, Claude, & Strimmer, 2004). The ‘splits’ package calculates the likelihood of the tree under a single coalescent (null model) and under a GMYC model, which may follow the single‐threshold or the multiple‐threshold assumptions. In the first, a single threshold is placed at every node in the tree, and the threshold at the maximum likelihood solution delimits the number of evolutionary units. This method has shown to display close correlation with the number of species in the tree (Fujisawa & Barraclough, 2013). The multiple‐threshold GMYC relaxes the assumption that speciation events must be older than all coalescent events in the gene tree. The method iteratively splits and fuses existing species clusters starting from the single‐threshold solution, until no further improvement in the maximum likelihood occurs (Fujisawa & Barraclough, 2013). We performed both and compared the results in terms of the number of OTUs delimited. Besides these tree‐based methods, we used two distance‐based approaches for delimiting the number of OTUs, namely ABGD (Automatic Barcode Gap Discovery) (Puillandre, Lambert, Brouillet, & Achaz, 2016) and jMOTU (Jones, Ghoorah, & Blaxter, 2011). ABGD uses genetic divergences between sequences and a coalescent model to identify a barcode gap between intra‐ and interspecific distances and defines OTUs accordingly (Puillandre et al., 2016). jMOTU takes one, or a range of, user‐supplied distance cutoff values and clusters sequences using single‐linkage clustering so that no member of one OTU is closer than the cutoff to any member of any other cluster (Jones et al., 2011). A detailed explanation of the basis and principles of each method is provided in Table 2 and Figure 2.

**Table 2:** Number of groups ABGD partition the dataset into with initial (IP) and recursive (RP) partitioning under different models (JC69 or K80), transition/transversion ratios (TS/TV), gap widths (*X*), and prior values on maximum intraspecific divergence

Partition:	RP	IP	RP	IP	RP	IP	RP	IP	RP	IP	RP	IP	RP	IP	RP	IP	RP	IP	RP	IP	RP	IP	RP	IP
*X*:	0.5	0.5	0.5	0.5	0.5	0.5	1.0	1.0	1.0	1.0	1.0	1.0	1.5	1.5	1.5	1.5	1.5	1.5	2.0	2.0	2.0	2.0	2.0	2.0
Model:	JC	JC	K80	K80	K80	K80	JC	JC	K80	K80	K80	K80	JC	JC	K80	K80	K80	K80	JC	JC	K80	K80	K80	K80
TS/TV:			2	2	4	4			2	2	4	4			2	2	4	4			2	2	4	4
Prior																								
0.005000	25	22	25	22	25	22	25	22	25	22	25	22	25	22	25	22	25	22	25	22	25	22	25	22
0.006458	24	22	24	22	24	22	24	22	24	22	24	22	24	22	24	22	24	22	24	22	24	22	24	22
0.008341	24	22	24	22	24	22	24	22	24	22	24	22	24	22	24	22	24	22	24	22	24	22	24	22
0.010772	23	22	23	22	23	22	23	22	23	22	23	22	23	22	23	22	23	22	21	17	21	17	21	17
0.013913	22	20	23	22	23	22	21	17	21	17	21	17	21	17	21	17	21	17	20	17	20	17	20	17
0.017969	20	17	20	17	20	17	20	17	20	17	20	17	20	17	20	17	20	17	20	17	20	17	20	17
0.023208	19	17	19	17	19	17	19	17	19	17	19	17	17	17	17	17	19	17	17	17	17	17	19	17
0.029974	17	17	17	17	17	17	17	17	17	17	17	17	17	17	17	17	17	17	17	17	17	17	17	17
0.038713	17	17	17	17	17	17	17	17	17	17	17	17	17	17	17	17	17	17	17	17	17	17	17	17
0.050000	17	17	17	17	17	17	17	17	17	17	17	17	17	17	17	17	17	17	NA	NA	NA	NA	17	17

Note

NA = not applicable parameter combination according to ABGD web version. Automatic barcode gap discovery (ABGD) uses genetic distances to identify a barcode gap between intra‐ and interspecific distances, but needs a prior on maximum intraspecific divergence. The prior is used to calculate an estimate of the population mutation rate parameter θ. This estimate is calculated as the average pairwise of all distances below the specified prior. The estimate of θ is used to set a distance limit above which there is a 5% risk of a barcoding gap being intraspecific, directly inferred from a coalescent model of a single nonstructured panmictic population. Only barcode gaps found above this distance limit are used to infer a partition. Gaps are inferred based on peak slope values of ranked ordered distances with a dynamic window size for calculating the slope. ABGD performs recursive partitioning, using the same prior, on each group from the initial partition until no further splitting occurs. Apart from the prior, which can be set to a range of values, the method requires parameter *X* to be specified which is the minimum gap width relative to any gap in the prior intraspecific divergence (default *X* = 1.5). *X* relates to the sensitivity of the method to the size of the barcoding gap relative to intraspecific gaps. Genetic distances were calculated under two models (JC69 and K80). Under the K80 model, we used two values (2 and 4) on the transition/transversion ratio (TS/TV) after this parameter was estimated for the dataset to 3.06 (95% HPD: 2.66–3.43) in a MrBayes 3.2.6 run under a HKY85 model with one million MCMC generations. We explored four values for *X* (0.5, 1.0, 1.5, and 2.0) and ten steps in prior space between 0.005 and 0.05. The ABGD analyses yielded very stable results under different models used to calculate distances. At prior maximum intraspecific divergence values from 0.03 and above, all analyses divided the dataset into 17 groups, corresponding to named species. At lower prior values (0.023–0.005) between 19 and 25 groups were delimited and the recursive partitioning function split the data into 2–3 additional groups compared to the primary partition. This is indication of a dataset with multiple distance thresholds throughout included taxa. Primary partitions make a jump from 22 to 17 delimited groups somewhere between a prior value of 0.008 and 0.018, depending on the gap width parameter. The recursive partitioning makes a smoother transition over this prior space with an intermediate number of delimited groups from 24 at 0.008 to 20 at 0.018. At the default value of *X* (1.5) and at a prior value of 0.01 indicated by Puillandre et al. (2016) as a suitable value to match species hypotheses based on independent data across a majority of tested empirical datasets, 23 groups were delimited. This partitioning scheme involved subdivision of Euproctis chrysorrhoea (2 groups), Peribatodes ilicaria (2 groups), *Ennomos quercaria* (2), Catocala nymphagoga (2 groups), and Colotois pennaria (3 groups). In the maximum division at the lowest prior value (0.005) also *Watsonalla uncinula* was subdivided into 2 groups and *Ennomos quercaria* into 3 separate groups. These 25 groups are identical to the 25 delimited units by single‐threshold GMYC except Tortricodes alternella is not subdivided and instead *Ennomos quercaria* forms three groups instead of two.

### 2.6 Statistical analyses

Our main goal was to assess whether the inclusion of sequences from genetic diversity hot spots (in our case the Iberian and Italian Peninsulas) increases intraspecific genetic divergence. We did not consider the Balkan Peninsula, as barely any sequences were available in BOLD and we had not sampled in that geographic region.

In order to test whether southern Europe was underrepresented in the Barcode of Life Data System relative to its species richness, we checked the geographical distribution of all the study species at the GBIF website (GBIF.org, 2017). The study species are common ones and we could thus assess their distribution range reliably on GBIF records. In parallel, we checked another database (Lepidoptera Mundi, lepidoptera.eu) based on records and bibliographical data to confirm the species geographical distribution. We took the southernmost and northernmost European records for each species of the study group and assumed that these were the limits of its geographical distribution in Europe; in between them, the species would be present. We then counted to which extent the number of species recorded decreased with increasing latitude starting from southern Iberian Peninsula. At the same time, and taking only the DNA barcodes available in BOLD (not including the individuals sequenced in this project), we assessed the relationship between latitude and the number of barcodes. Regression fitting was done using STATISTICA (Statoft Inc, 2005).

To assess whether genetic divergence was higher in the pairwise interpopulation comparisons when at least one of the populations was Iberian or Italian, we performed linear mixed models (LMMs) using ‘nlme’ package (Pinheiro, Bates, De Roy, & Sarkar, 2017) of R (R Core Team, 2016). We did so because we considered both fixed and random mixed effects in the regression models. Four types of pairwise contrasts between populations were defined as follows: (a) between two European populations excluding Iberian and Italian ones (contrasts abbreviated henceforth as EUEU), (b) between one European population (not Italian) and one Iberian (abbreviation EUIB), (c) between one European population (not Iberian) and one Italian (EUIT), and (d) between two Iberian populations (IBIB). The pairwise comparisons between only Italian populations were not conducted due to low sample size.

We performed three LMM tests: the first one to assess whether the genetic divergence differed between EUEU and EUIB pairwise population contrasts; the second to calculate the same but between EUEU and EUIT; and the third one to assess it within the same geographical area (EUEU vs. IBIB contrasts). In all the analyses, the genetic divergence (measured as K2P% distance) was the dependent variable and the type of population contrast the independent factor; the pairwise spatial distance between populations was the covariate. Additionally, the largest number of sequences at each pairwise comparison between populations was also included as covariate to control for the potential effect that sample size could have on genetic divergence. In the EUEU versus IBIB analysis, the spatial range was reduced to 1,000 km, as the maximum distance between any pair of Iberian populations was lower than that. In the three tests, the species of Lepidoptera was included as a random factor.

## 3 RESULTS

### 3.1 Geographical distribution of DNA barcodes in Europe

The geographical distribution of the DNA barcodes of the study species showed a strong bias with an underrepresentation of southern Europe. If the barcodes available in BOLD are divided between those from countries located at southern European Peninsulas (Italy, Iberia, and the Balkans) and the rest of the continent, the number is clearly imbalanced (73 vs. 204). Germany is the country in which most DNA barcodes are available (60), followed by some of its neighbor countries (Austria, Czech Republic, France, and the Netherlands) with more than 15 each (Table 3). The highest number in the northern countries is not related to their area.

**Table 3:** Number of DNA barcodes of studied species available in BOLD by country

Country	N_Barcodes
Austria	16
Croatia^a^	1
Czech Republic	24
Finland	20
France	20
Germany	60
Greece^a^	1
Italy^a^	41
Macedonia	2
Netherlands	22
Romania	10
Slovenia	1
Spain^a^	30
United Kingdom	29

^a^ The countries that belong to the southern Europe peninsulas.

**Figure 3:**
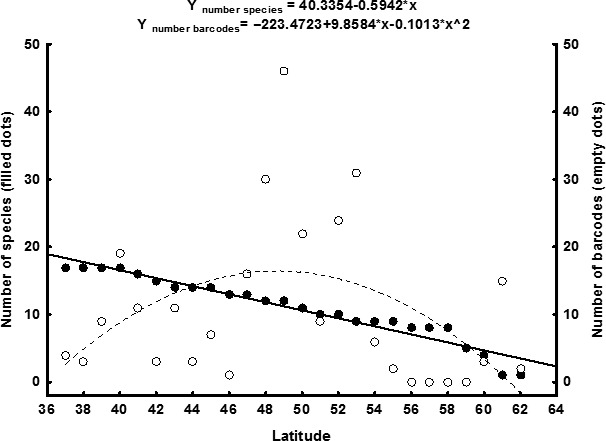
Number of species (filled dots, continuous line) and number of barcodes (empty dots, broken line) recorded along the European latitudinal (degrees) gradient analyzed in this study. Regression equations for both variables are show on the top. The *R* values for the regression models were *R* = 0.45 for the number of barcodes and *R* = 0.97 for the number of species.

The species richness does not explain the geographical bias in DNA barcodes either. Taking our study species as a model, the number of species decreases significantly in a linear fashion from south to north (*y* = 40.3354 − 0.5942*x*) (*F*_1,24_ = 393.44; *p* < .001. Figure 3). By contrast, the relationship between the number of barcodes and latitude is quadratic (*y* = −223.4723 + 9.8584*x* − 0.1013*x* ^2^) (*F*_3,25_ = 10.16; *p* < .001. Figure 3). By equaling to zero the derivative of the previous function (*y*′ = 9.8584 − 0.2026*x*), we found that the number of barcodes peaked at 48.66°, and it increased from the south to that latitude and decreased northwards (Figure 3). When the functions relating latitude with species richness and number of DNA barcodes are plotted together, it can be seen that, in latitudes lower than 44°, the number of DNA barcodes is proportionally low compared to the number of species (Figure 3). This leaves the Iberian Peninsula (with a highest latitude for a DNA barcode in BOLD of 42.74°N), among the underrepresented areas, as well as most parts of the other two peninsulas of southern Europe (Italy and the Balkans) (Figure 3).

### 3.2 DNA barcode geographical bias and intraspecific genetic diversity

The number of Iberian haplotypes recorded in this study added up to 109, from which 84 (77%) were new for BOLD. In all cases, the morphological identification of the caterpillars was coincident with the closest species match in BOLD. However, in three species, the intraspecific K2P distance with respect any reference barcode exceeded 1% (Table 4), what is over the maximum BOLD strict threshold for unequivocal species assignment. After pooling together the DNA sequences available in BOLD with those obtained from the new specimens sampled in Iberia, the total number of barcodes scaled up to 652. The new sequences also increased the number of Iberian localities with DNA barcodes, thereby allowing more pairwise comparisons with non‐Iberian Europe. The species‐by‐species comparisons among the Europe–Europe, Europe–Italy, Europe–Iberia, and Iberia–Iberia contrasts (EUEU, EUIT, EUIB, and IBIB, respectively) showed that the inclusion of Iberian samples increased the intraspecific genetic divergence (not considering spatial distance) (Table 1). In all cases except one (*Orthosia cruda*), the maximum genetic divergence was higher in EUIB comparisons than in EUEU ones. In the case of Italy, the effect was not so strong, as the genetic divergence in Europe–Europe and Europe–Italy contrasts (EUEU and EUIT, respectively) was very similar (Table 1). Only in some cases (e.g. *Archips xylosteana*), the maximum genetic divergence was higher in EUIT comparisons than in EUEU ones. The EUEU and IBIB contrasts retrieved the same divergent results: maximum pairwise intraspecific genetic divergence between Iberian populations was much higher, and almost reached 2% in 7 out of 18 species (*Catocala nymphagoga*,* Peribatodes ilicaria*,* Ennomos quercaria*,* Tortricodes alternella*,* Watsonalla uncinula*,* C. pennaria*, and* Euproctis chrysorrhoea*) (Table 1). These differences are particularly striking, since the maximum pairwise spatial distance between populations was lower in Iberia compared to the rest of Europe (852.63 km vs. 2,476.70 km).

**Table 4:** Total number of haplotypes per species used in the analyses, number of haplotypes recorded in Iberia in the present study, and number of these Iberian haplotypes new to the Barcode of Life Data Systems BOLD (within brackets, number of new haplotypes that could not be identified in BOLD because the genetic distance exceeded 1% with respect to any reference barcode)

Species	Total Haplotypes	Haplotypes Iberian sampling	New haplotypes
*Archips xylosteana*	6	2	1
*Bena bicolorana*	12	3	2
*Catocala nymphagoga*	15	11	9
*Colotois pennaria*	4	2	2
*Colotois pennaria carbonii*	9	0	0
*Dryobotodes eremita*	20	15	12
*Dryobotodes monochroma*	16	15	11
*Ennomos quercaria*	6	3	2 (1)
*Eupithecia cocciferata*	6	4	3
*Euproctis chrysorrhoea*	8	0	0
*Malacosoma neustria*	10	5	2
*Nycteola columbana*	7	7	4
*Orthosia cruda*	16	1	1
*Peribatodes ilicaria*	12	10	9 (3)
*Tortricodes alternella*	20	16	16 (16)
*Tortrix viridana*	12	8	3
*Watsonalla uncinula*	6	3	3
*Xanthia ruticilla*	10	4	4

**Figure 4:**
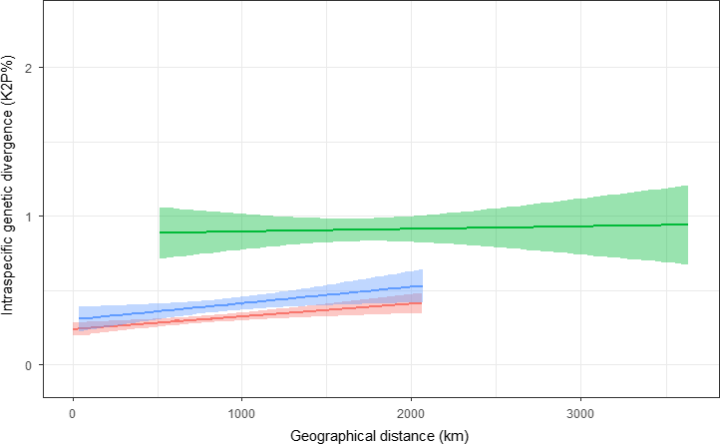
Relationship between geographic distance (*x*‐axis, km) and intraspecific genetic divergence (*y*‐axis, K2P%) (Mean ± SD) in pairwise contrasts between European and Iberian populations (EUIB, green line), between European populations (EUEU, red line), and between European and Italian populations (EUIT, blue line)

**Figure 5:**
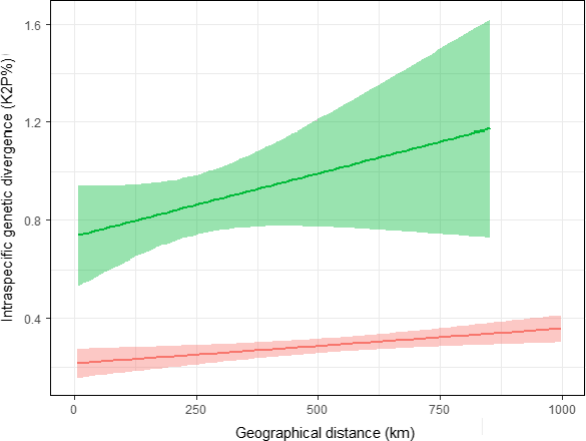
Relationship between geographic distance (*x*‐axis, km) and intraspecific genetic divergence (*y*‐axis, K2P%) (Mean ± SD) in pairwise contrasts between Iberian populations (IBIB, green line) and only European populations (EUEU, red line)

The higher intraspecific genetic divergence in the pairwise comparisons including Iberian populations (EUIB and IBIB) held true also when the spatial distance was included as a covariate. In the analyses comparing EUEU versus EUIB contrasts, the distance between localities had a significant positive effect on genetic divergence (Table 5; Figure 4). However, the type of contrast (EUEU or EUIB) had an independent and significant effect too as, for any pairwise spatial distance, the average genetic divergence was higher if one of the two populations compared was Iberian (EUIB divergence higher than EUEU) (Table 5; Figure 4). The same happened in the analyses of EUEU versus IBIB over a maximum range of 1,000 km. The genetic divergence between Iberian populations (IBIB) was higher than in the pairwise comparisons between European ones (EUEU) independently of the effect of the geographical distance between localities (Table 5; Figure 5). In the analyses comparing EUEU versus EUIT contrasts, the distance between localities had a significant positive effect on genetic divergence too (Table 5; Figure 4). The effect of including Italian populations was not as high as in the case of Iberia, but the pairwise genetic divergence was higher between EUIT contrasts than between EUEU ones (Table 5; Figure 4). As expected, the maximum sample size of each pairwise comparison had a significant effect in all LMMs but, after controlling for it, the type of contrast remained highly significant in all cases.

**Table 5:** Results of the Linear Models testing the effects on the intraspecific genetic distance (dependent variable) of the pairwise spatial distance between populations (Dis.Geo, covariate) and the type of pairwise geographic comparison between populations (Compar, independent factor), considering the effect of maximum sample size (Nmax, covariate). Statistically significant results are shown in bold

EUEU‐EUIB	*F* (1, 1,299)	*P*
Comp.	336.098	**<.001**
Dis.Geo	18.536	**<.001**
Nmax	51.044	**<.001**

EUEU‐EUIT	*F* (1, 1,090)	*P*
Comp.	8.415	**<.001**
Dis.Geo	49.171	**<.001**
Nmax	23.705	**<.001**

EUEU‐IBIB	*F* (1, 994)	*P*
Comp.	122.191	**.002**
Dis.Geo	19.860	**<.001**
Nmax	13.284	**<.001**

### 3.3 Intraspecific genetic divergence and biogeography

**Figure 6:**
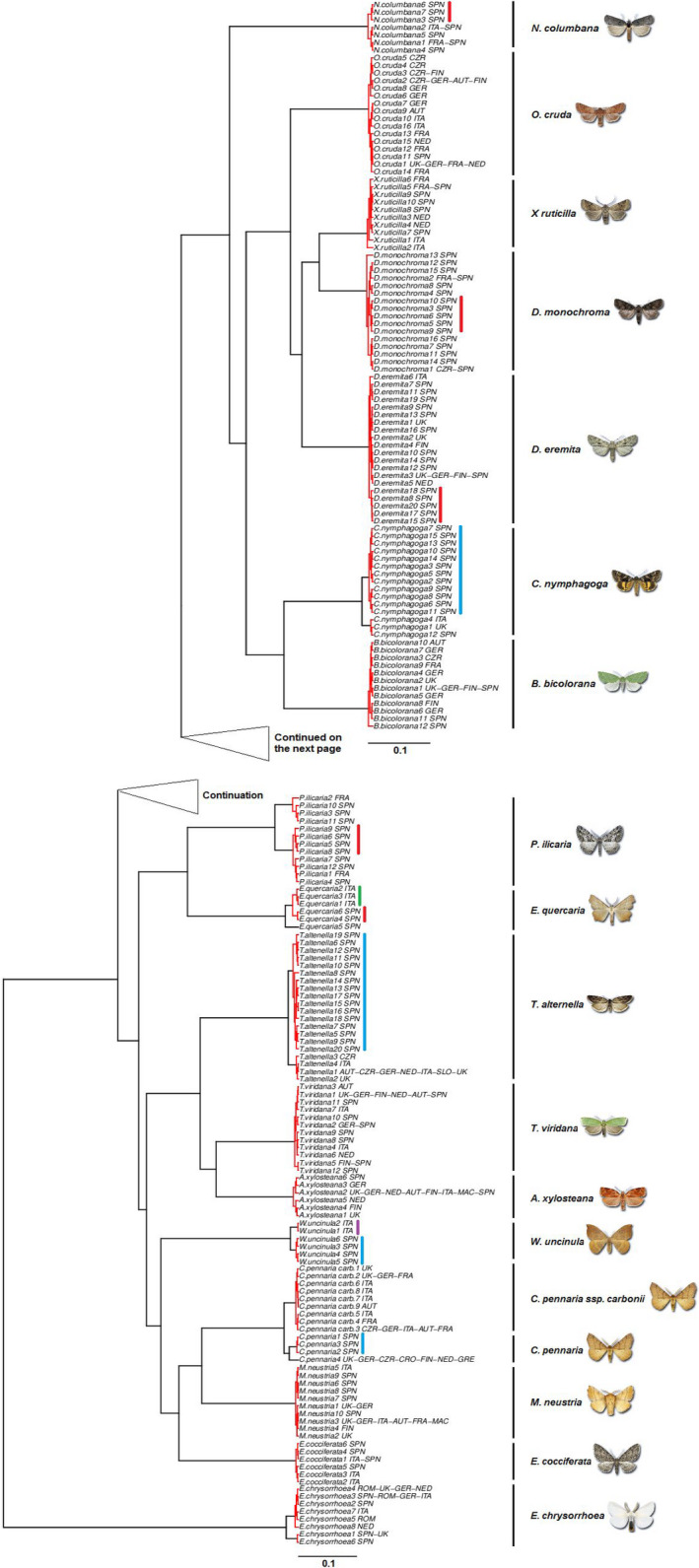
Ultrametric gene tree including all COI haplotypes used for species delimitation with the GMYC model. Red branch splits show the intraspecific divergence within clusters that correspond to different OTUs (operational taxonomic units) or putative species. Black lines and Linnean species names on the right indicate the species identity according to morphological determination. Red lines show monophyletic Iberian clades within recognized Linnean species. Blue lines indicate Iberian clusters classified as different putative species by GMYC. Green line (*Ennomos quercaria*) shows monophyletic Italian clade within recognized Linnean species. Purple line (*Watsonalla uncinula*) indicates Italian cluster classified as different putative species by GMYC. The letters besides the scientific name in the taxon labels indicate the country/ies in which each haplotype was recorded (AUT, Austria; CRO, Croatia; CZR, Czech Republic; FIN, Finland; FRA, France; GER, Germany; GRE, Greece; ITA, Italy; NED, Netherlands; MAC, Macedonia; ROM, Romania; SLO, Slovenia; SPN, Spain; and UK, United Kingdom)

The species delimitation using the GMYC single‐threshold model defined 25 operational taxonomic units (OTUs) or putative species (Table 6), of which 18 corresponded to recognized Linnean species or (1) subspecies (Figure 6). The rest correspond to Iberian haplotype clusters classified as independent OTUs mostly within those species with a maximum intraspecific genetic divergence over 2% (Table 1), namely *C. nymphagoga*, *C. pennaria*,* T. alternella*, and *W. uncinula*. In addition, within *Dryobotodes eremita*,* Dryobotodes monochroma*,* E. quercaria*,* Nycteola columbana*, and* P. ilicaria*, there were well‐defined Iberian monophyletic clades (although not delimited at separate OTUs in the GMYC analysis). This Iberian distinctiveness with respect to the rest of Europe was higher than in the case of Italy. There were fewer Italian OTUs/monophyletic clades and more haplotypes were shared between Italy and the countries north of the Alps than between Iberia and the rest of the continent (Figure 6). Haplotype sharing is also frequent between populations located in the central and northern European countries (Figure 6).

**Table 6:** Subdivided taxa and number of operational taxonomic units defined by GMYC (single and multiple threshold), jMOTU (at a cutoff value of 1.1%) and ABGD (at a 0.01 prior on maximum intraspecific divergence and gap width (*X*) as default) See Table 6 and Figure 2 for an exploration of the effect of different parameter values in ABGD and jMOTU analyses

Species	GMYCs	jMOTU	ABGD	GMYCm
*Catocala nymphagoga*	2	2	2	3
*Peribatodes ilicaria*	2	2	2	4
*Euproctis chrysorrhoea*	2	2	2	3
*Colotois pennaria*	3	3	3	3
*Ennomos quercaria*	2	2	2	3
*Watsonalla uncinula*	2	2	1	2
*Tortricodes alternella*	2	2	1	2
*Nycteola columbana*	1	1	1	2
*Orthosia cruda*	1	1	1	3
*Xanthia ruticilla*	1	1	1	2
*Dryobotodes monochroma*	1	1	1	2
*Archips xylosteana*	1	1	1	2
Total number of OTUs	25	25	23	36

**Figure 7:**
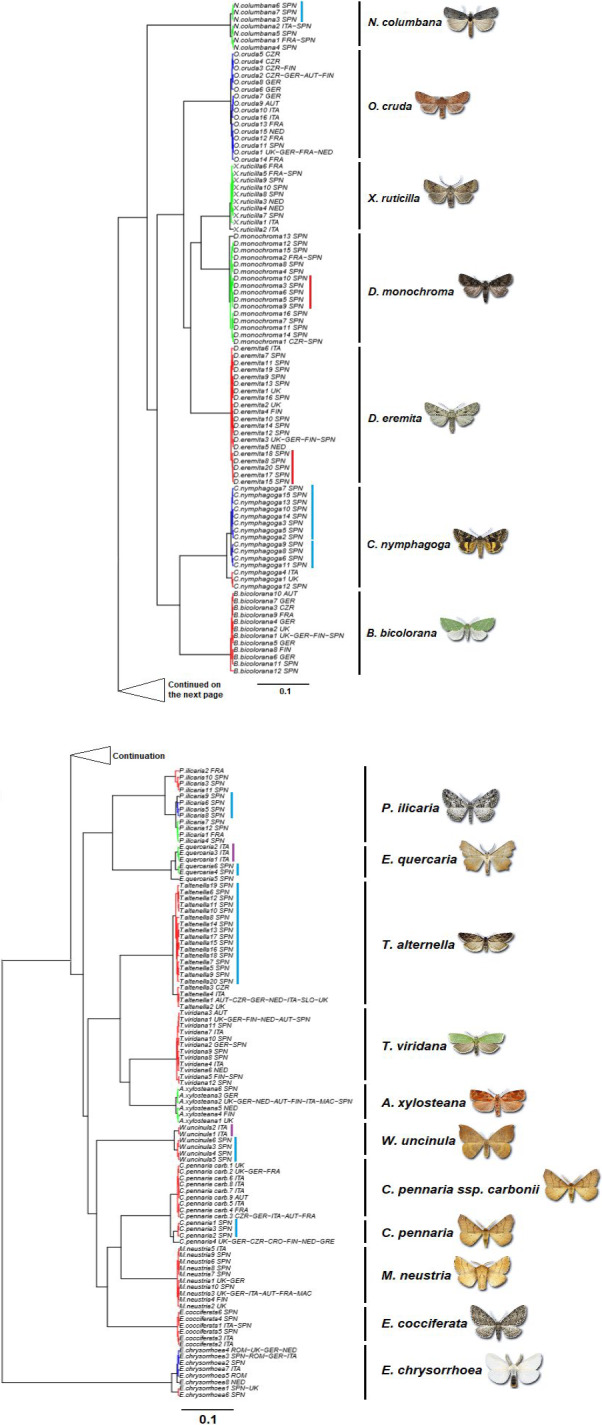
Ultrametric gene tree including all COI haplotypes used for species delimitation with the multiple‐threshold GMYC model. Red (first threshold), blue (second threshold), and green (third threshold) branches show the intraspecific divergence within clusters that correspond to different OTUs (operational taxonomic units) or putative species. Black lines and Linnean species names on the right indicate the species identity according to morphological determination. Red lines show monophyletic Iberian clades within recognized Linnean species. Blue lines indicate Iberian clusters classified as different putative species by GMYC. Purple line (*Watsonalla uncinula*) indicates Italian cluster classified as different putative species by GMYC. The letters besides the scientific name in the taxon labels indicate the country/ies in which each haplotype was recorded (AUT, Austria; CRO, Croatia; CZR, Czech Republic; FIN, Finland; FRA, France; GER, Germany; GRE, Greece; ITA, Italy; NED, Netherlands; MAC, Macedonia; ROM, Romania; SLO, Slovenia; SPN, Spain; UK, United Kingdom)

The number of putative species delimited by the multiple‐threshold GMYC largely exceeded that of the single‐threshold model (Table 6). All the putative species delimited by the latter were also different OTUs according to the former. However, according to the multiple‐threshold GMYC, more Iberian monophyletic clades were classified as different putative species (Figure 7). Both ABGD and jMOTU produced very similar result to the GMYC single‐threshold approach, both in terms of the number and in terms of identity of the OTUs retrieved (Table 6). jMOTU delimited the exact same 25 OTUs as single‐threshold GMYC at a cutoff value of 7 bp (equivalent to 1.1% genetic divergence) (Table 6; Figure 2). ABGD delimited 23 of the same 25 OTUs at a recommended prior value on maximum intraspecific divergence of 0.01, the sole difference being no subdivision of *T. alternella* and *W. uncinula* (Tables 2 and 6).

## 4 DISCUSSION

The number of DNA barcodes for oak‐feeding Lepidoptera is lower in southern Europe, despite the higher species richness. As expected, the effect of the geographical scale on the genetic divergence depended on the latitude. In pairwise sequence comparisons, for any given spatial distance the genetic divergence was higher when at least one of the sequences came from one of the southern European peninsulas included in the study (Italy and Iberia). This made identification of some southern query sequences problematic, as the genetic distance with respect to the reference barcodes in BOLD was above the maximum intraspecific threshold allowed. The effect of the latitude is due to the presence of southern haplotypes with a reduced geographical distribution. Accordingly, the COI gene tree showed in different species monophyletic clades exclusive from southern Europe (mainly Iberian). The GMYC single‐threshold model classified some of those clusters as different OTUs and thus potentially cryptic species.

The lower availability of DNA barcodes in southern Europe cannot be explained by any factor but the regional scarcity of DNA barcoding initiatives. Before we started the project, only 26.36% of the DNA barcodes available for the study species in BOLD came from the southern European Peninsulas (Iberia, Italy, and the Balkans). The Iberian Peninsula was underrepresented according to its area and to its species richness. In fact, for some species (*A. xylosteana*, *Bena bicolorana*, *D. eremita*, *D. monochroma*, *E. quercaria*, *Eupithecia cocciferata*, *Malacosoma neustria*, *N. columbana*, *O. cruda*, *T. alternella*, and *Tortrix viridana*), no Iberian sample had ever been sequenced before the present study (January 2018) and others, restricted to the southwestern Mediterranean Basin or Iberia (*D. labecula*, *P. torrenti*), were sequenced for the first time. The function relating latitude and number of barcodes peaked at 48 degrees north, because that is the latitude around which the largest European barcoding initiative has been carried out in Germany (Gemeinholzer et al., 2011).

Previous studies have shown that the larger the sampling scale, the greater the intraspecific divergence and the more likely finding overlapping closely related taxa (Bergsten et al., 2012). In this study, we did not analyze the effects on the barcoding gap, because the closest relatives of the study species often feed on other host plants (Cates, 1981; Thompson & Pellmyr, 1991). Rather, we focused on intraspecific genetic divergence but considering not only the effect of the spatial distance alone, but its interaction with the latitude. Doing so we found that intraspecific genetic divergence was higher in pairwise comparisons that included at least one DNA sequence from a southern peninsula than when both came from elsewhere in the continent. The peninsulas of southern Europe are hot spots of species and genetic diversity (Geiger et al., 2014; Murienne & Giribet, 2009; Pinto et al., 2012); thus, when they are undersampled, intraspecific genetic divergence is underestimated more than expected by the mere reduction of the geographical scale. The low availability of DNA barcodes or their reduced geographic distribution is a main concern in DNA barcoding (Bergsten et al., 2012; Dincă et al., 2015; Geiger et al., 2014; Savolainen et al., 2005). Our results show that, to capture as much intraspecific genetic variability as possible, sequencing efforts should be concentrated in southern European genetic diversity hot spots (Dincă et al., 2015; Murienne & Giribet, 2009; Pinto et al., 2012) where, paradoxically, the number of available DNA barcodes is lower.

The disproportionately strong effect of the Iberian samples is largely related with the distribution patterns of genetic diversity determined by Pleistocene glaciations (Hewitt, 1996; Schmitt, 2007). The southern European Peninsulas were refugia that hosted a large number of plant and animal taxa when the ice sheet covered large extensions of the continent. This was the case of our study group (insects associated with oaks and other species of broad‐leaved trees). In the Iberian Peninsula, where a greater geographic isolation is observed than, for example, in the Italian peninsula, deciduous and evergreen oak forests were restricted to a few refugia close to the coast or at the south‐facing slopes of some mountainous systems (Koster, 2005; Magri et al., 2007). When the ice retreated, not all haplotypes spread northwards but just some of them. This “founder effect” is responsible for the higher species richness in the south and the genetic homogeneity of the recently colonized areas in the central and northern parts of the continent (Hewitt, 1996, 1999; Taberlet, Fumagalli, Wurst‐Saucy, & Cosson, 1998). Taking our study species as an example, there are many examples of haplotypes shared by different central and northern European countries, especially between Germany, the Netherlands, the United Kingdom, Czech Republic, Austria, or Finland. Similarly, a noteworthy study including hundreds of Lepidoptera species showed little intraspecific genetic variability between central (Austria) and northern (Finland) in Europe (Huemer et al., 2014).

Due to the historical factors linked to the paleoclimate of the continent, in 56% of our study species, we found monophyletic Iberian clades. This was not the case for the Italian peninsula, which shared a higher genetic similarity with the territories northwards, suggesting a stronger effect of the Pyrenees as geographical barrier than that of the Alps. Previous studies have reported similar results for organisms like butterflies or freshwater fish (Dincă et al., 2015; Geiger et al., 2014). Some alpine butterflies, for example, show higher intraspecific genetic distance among the populations in the Alps and the nearby Pyrenees than among the Alps and Scandinavia (Dincă et al., 2015). However, it is well known that the Alps have a deep impact on the genetic variation of other groups of animals (Arntzen, 2001; Cornetti et al., 2016; Leys, Keller, Räsänen, Gattolliat, & Robinson, 2016).

Four Iberian clades were retrieved as different OTUs by the GMYC single‐threshold model, which confirmed its utility for species delimitation within poorly inventoried biogeographic regions in Europe. According to Fujisawa and Barraclough (2013), GMYC single‐threshold model is more reliable than the multiple thresholds one, which overestimates the number of OTUs. Moreover, ABGD and jMOTU retrieved identical or very similar results to the single‐threshold GMYC, while the latter was considered the most reliable of the three in a comparison of performances (Ratnasingham & Herbert, 2013).

The presence of different putative species in southern Europe conditions the efficacy of species identification on the basis of DNA barcoding (Derkarabetian & Hedin, 2014; Dincă et al., 2015; Fossen, Ekrem, Nilsson, & Bergsten, 2016; Geiger et al., 2014). In the hypothetical case that there had not been any Iberian barcode in BOLD, in 7 out of 15 species (possible comparisons between Iberia and Europe, Table 2), there would have been at least one haplotype that would have not been determined to the species level (EUIB K2P distance above 1%). Even including the Iberian barcodes available in BOLD before the present study, the same still happened in three species. The case of *T. alternella* is specially remarkable: from 16 new haplotypes recorded in this study, none of them could not be matched to any reference sequence in BOLD using the 1% threshold (Table 4). This lack of identification due to the absence of Iberian reference sequences is not exclusive of the study species, having been reported for other insect taxa as well (e.g., *Cerambyx cerdo*, Coleoptera) (Torres‐Vila & Bonal, 2019).

If well the present dataset shows a clear trend, we have to be cautious before generalizing, as it is restricted to a limited number of species of oak‐feeding moths. The occurrence of putative cryptic species in southern Europe in other taxa (Dincă et al., 2015; Geiger et al., 2014) suggests that the pattern may be widespread, but further studies are needed to confirm it. Future large‐scale DNA barcoding initiatives in Europe should cover latitudinal gradients, rather than large distances at the same latitude, to avoid neglecting genetic diversity hot spots like the Iberian Peninsula.

## CONFLICT OF INTEREST

The authors declare that there is no conflict of interests regarding the publication of this article.

## AUTHOR CONTRIBUTIONS

**Álvaro Gaytán:** Conceptualization (equal); Data curation (lead); Formal analysis (equal); Funding acquisition (equal); Investigation (lead); Methodology (lead); Project administration (equal); Resources (equal); Software (lead); Supervision (equal); Validation (equal); Visualization (equal); Writing‐original draft (equal); Writing‐review & editing (equal). **Johannes Bergsten:** Conceptualization (supporting); Data curation (supporting); Formal analysis (equal); Funding acquisition (supporting); Investigation (supporting); Methodology (equal); Project administration (supporting); Resources (equal); Software (lead); Supervision (equal); Validation (equal); Visualization (equal); Writing‐original draft (equal); Writing‐review & editing (equal). **Tara Canelo:** Data curation (supporting); Investigation (supporting); Resources (supporting). **Carlos Pérez‐Izquierdo:** Investigation (supporting); Resources (supporting). **Maria Santoro:** Data curation (equal); Resources (equal). **Raul Bonal:** Conceptualization (lead); Formal analysis (equal); Funding acquisition (lead); Investigation (equal); Methodology (equal); Project administration (lead); Resources (equal); Software (equal); Supervision (lead); Writing‐original draft (equal); Writing‐review & editing (equal).

## Supporting information

Supplementary Material

## Data Availability

The new sequences provided in this paper are available in the public repository BOLD (Citation: Gaytan et al., 2020. COI sequences of Iberian oak herbivores. BOLD systems. https://doi.org/10.5883/DS‐GEA1). The R‐Script and the alignment of the sequences used for the analyses are uploaded to the public repository GitHub (Citation: Gaytan et al., 2020. R‐script and alignment. GitHub. URL: https://github.com/alvarogaytan/Gaytanetal). The original specimens are stored at University of Extremadura entomological collection, Plasencia, Extremadura (Spain).

## References

[ece36733-bib-0001] Ahrens, D. , Fabrizi, S. , Šípek, P. , & Lago, P. K. (2013). Integrative analysis of DNA phylogeography and morphology of the European rose chafer (*Cetonia aurata*) to infer species taxonomy and patterns of postglacial colonisation in Europe. Molecular Phylogenetics and Evolution, 69, 83–94. 10.1016/j.ympev.2013.05.016 23727596

[ece36733-bib-0002] Arntzen, J. W. (2001). Genetic variation in the Italian crested newt, *Triturus carnifex*, and the origin of a non‐putative population north of the Alps. Biodiversity Conservation, 10, 971–987.

[ece36733-bib-0003] Avise, J. C. (2000). Phylogeography: The history and formation of species. Cambridge, MA: Harvard University Press.

[ece36733-bib-0004] Beentjes, K. , Speksnijder, A. , Van der Hoorn, B. , & Van Tol, J. (2015). DNA barcoding program at Naturalis Biodiversity Center, the Netherlands. Genome, 58, 193.

[ece36733-bib-0005] Bergsten, J. , Bilton, D. T. , Fujisawa, T. , Elliott, M. , Monaghan, M. T. , Balke, M. , … Vogler, A. P. (2012). The effect of geographical scale of sampling on DNA barcoding. Systematic Biology, 61, 851–869. 10.1093/sysbio/sys037 22398121 PMC3417044

[ece36733-bib-0006] Berthier, K. , Chapuis, M. P. , Moosavi, S. M. , Tohidi‐Esfahani, D. , & Sword, G. A. (2011). Nuclear insertions and heteroplasmy of mitochondrial DNA as two sources of intra‐individual genomic variation in grasshoppers. Systematic Entomology, 36, 285–299. 10.1111/j.1365-3113.2010.00561.x

[ece36733-bib-0007] Bonal, R. , Muñoz, A. , & Vogler, A. P. (2011). Complex selection on life history traits and the maintenance of variation in exaggerated rostrum length in acorn weevils. Oecologia, 167, 1053–1061. 10.1007/s00442-011-2036-7 21674207

[ece36733-bib-0008] Bonal, R. , Vargas‐Osuna, E. , Mena, J. D. , Aparicio, J. M. , Santoro, M. , & Martín, A. (2018). Looking for variable molecular markers in the chestnut gall wasp *Dryocosmus kuriphilus*: First comparison across genes. Scientific Reports, 8, 5631 10.1038/s41598-018-23754-z 29618725 PMC5884851

[ece36733-bib-0009] Camus, A. (1936–1954). Les chênes, Monographie du genere Quercus et Monographie du genre Lithocarpus. Encyclopédie Economique de Sylviculture. Vol. VI, VII, VIII. Paris, France: Editions Lechevalier.

[ece36733-bib-0010] Cates, R. G. (1981). Host plant predictability and the feeding patterns of monophagous, oligophagous and polyphagous insect herbivores. Oecologia, 48, 319–326. 10.1007/BF00346488 28309746

[ece36733-bib-0011] Collins, R. A. , & Cruickshank, R. H. (2012). The seven deadly sins of DNA barcoding. Molecular Ecology Resources, 13, 969–975. 10.1111/1755-0998.12046 23280099

[ece36733-bib-0012] Cornetti, L. , Lemoine, M. , Hilfiker, D. , Morger, J. , Reeh, K. , & Tschirren, B. (2016). Higher genetic diversity on mountain tops: The role of historical and contemporary processes in shaping genetic variation in the bank vole. Biological Journal of the Linnean Society, 118, 233–244. 10.1111/bij.12723

[ece36733-bib-0013] Derkarabetian, S. , & Hedin, M. (2014). Integrative taxonomy and species delimitation in harvestmen: A revision of the western North American genus Sclerobunus (Opiliones: Laniatores: Travunioidea). PLoS One, 9, e104982. 25144370 10.1371/journal.pone.0104982 PMC4140732

[ece36733-bib-0014] Dincă, V. , Montagud, S. , Talavera, G. , Hernández‐Roldán, J. , Munguira, M. L. , García‐Barros, E. , … Vila, R. (2015). DNA barcode reference library for Iberian butterflies enables a continental‐scale preview of potential cryptic diversity. Scientific Reports, 5, 12395 10.1038/srep12395 26205828 PMC4513295

[ece36733-bib-0015] Drummond, A. J. , Suchard, M. A. , Xie, D. , & Rambaut, A. (2012). Bayesian phylogenetics with BEAUti and the BEAST 1.7. Molecular Biology and Evolution, 29, 1969–1973. 10.1093/molbev/mss075 22367748 PMC3408070

[ece36733-bib-0016] Dubey, S. , Michaux, J. , Brünner, H. , Hutterer, R. , & Vogel, P. (2009). False phylogenies on wood mice due to cryptic cytochrome‐b pseudogene. Molecular Phylogenetics and Evolution, 50, 633–641. 10.1016/j.ympev.2008.12.008 19126432

[ece36733-bib-0017] Edgard, R. C. (2004). MUSCLE: Multiple sequence alignment with highly accuracy and high throughput. Nucleic Acids Resources, 32, 1792–1797. 10.1093/nar/gkh340 PMC390337 15034147

[ece36733-bib-0018] Ekrem, T. , Alsos, I. G. , Willassen, E. , Johnsen, A. , Kleven, O. , Hobæk, A. , … Aakra, K. (2015). The Norwegian barcode of life network (NorBOL). Genome, 58, 214.

[ece36733-bib-0019] Elkinton, J. S. , Healy, W. M. , Buonaccorsi, J. P. , Boettner, G. H. , Hazzard, M. , & Smith, H. R. (1996). Interactions among gypsy moths, white‐footed mice, and acorns. Ecological Society of America, 77, 2332–2342. 10.2307/2265735

[ece36733-bib-0020] Ezard, T. , Fujisawa, T. , & Barraclough, T. G. (2009). SPLITS: SPecies' LImits by threshold statistics. R package version 1.0‐18/r45. Retrieved from http://R‐Forge.R‐project.org/projects/splits/

[ece36733-bib-0021] Fibiger, M. (1997). Noctuidae Europaeae, Vol. 3: Noctuinae III. Mitteilungen‐schweizerische Entomologische Gesellschaft.

[ece36733-bib-0022] Folmer, O. , Black, M. , Hoeh, W. , Lutz, R. , & Vrijenhoek, R. (1994). DNA primers for amplification of mitochondrial cytochrome c oxidase subunit I diverse metazoan invertebrates. Molecular Marine Biology and Biotechnology, 3, 294–299. 7881515

[ece36733-bib-0023] Fossen, E. I. , Ekrem, T. , Nilsson, A. N. , & Bergsten, J. (2016). Species delimitation in northern European water scavenger beetles of the genus *Hydrobius* (Coleoptera, Hydrophilidae). ZooKeys, 564, 71–120. 10.3897/zookeys.564.6558 PMC4820092 27081333

[ece36733-bib-0024] Führer, E. (1998). Oak decline in Central Europe: A synopsis of hypotheses In McManus M. L., & Liebhold A. M. (Eds.), Proceedings: Population dynamics, impacts, and integrated management of forest defoliating insects (pp. 7–24). USDA Forest Service General Technical Report NE.

[ece36733-bib-0025] Fujisawa, T. , & Barraclough, T. (2013). Delimiting species using single‐locus data and the generalized mixed yule coalescent approach: A revised method and evaluation on simulated data sets. Systematic Biology, 62, 707–724. 10.1093/sysbio/syt033 23681854 PMC3739884

[ece36733-bib-0026] Gaytan, A. , Bergsten, J. , Canelo, T. , Pérez‐Izquierdo, C. , Santoro, M. , & Bonal, R. (2020). COI sequences of Iberian oak herbivores. BOLD systems. 10.5883/DS-GEA1

[ece36733-bib-0027] Gaytán, A. , Canelo, T. , González‐Bornay, G. , Pérez‐Izquierdo, C. , & Bonal, R. (2018). Guía y clave de identificación de las orugas de los lepidópteros defoliadores del arbolado de la dehesa. Madrid, Spain: Ministerio de Agricultura, Pesca y Alimentación.

[ece36733-bib-0028] GBIF.org. (2017). GBIF Home Page. Retrieved from http://GBIF.org

[ece36733-bib-0029] Geiger, M. F. , Herder, F. , Monaghan, M. T. , Almada, V. , Barbieri, R. , Bariche, M. , … Freyhof, J. (2014). Spatial heterogeneity in the Mediterranean Biodiversity Hotspot affects barcoding accuracy of its freshwater fishes. Molecular Ecology Resources, 14, 1210–1221. 10.1111/1755-0998.12257 24690331

[ece36733-bib-0030] Gemeinholzer, B. , Dröge, G. , Zetzsche, H. , Haszprunar, G. , Klenk, H.‐P. , Güntsch, A. , … Wägele, J.‐W. (2011). The DNA Bank Network: The start from a German initiative. Biopreservation Biobank, 9, 51–55. 10.1089/bio.2010.0029 24850206

[ece36733-bib-0031] Goater, B. , Ronkay, L. , & Fibiger, M. (2003). Noctuidae Europaeae, Catocalinae and Plusiinae, vol. 10 Soro, Denmark: Entomology Press, 452.

[ece36733-bib-0032] Gomez de Aizupura, C. (2002). Orugas y mariposas de Europa: Orden Lepidoptera. Hetrocera: lepidópteros de actividad nocturna. Organismo Autónomo de Parques Nacionales.

[ece36733-bib-0033] Gunay, F. , Alten, B. , Simsek, F. , Aldemir, A. , & Linton, Y. M. (2015). Barcoding Turkish *Culex* mosquitoes to facilitate arbovirus vector incrimination studies reveals hidden diversity and new potential vectors. Acta Tropica, 143, 112–120. 10.1016/j.actatropica.2014.10.013 25446171

[ece36733-bib-0034] Hajibabaei, M. , Singer, G. A. C. , Hebert, P. D. N. , & Hickey, D. A. (2007). DNA barcoding: How it complements taxonomy, molecular phylogenetics and population genetics. Trends Genetics, 23, 167–172. 10.1016/j.tig.2007.02.001 17316886

[ece36733-bib-0035] Hebert, P. D. N. , Cywinska, A. , Ball, S. L. , & de Waard, J. R. (2003). Biological identifications through DNA barcodes. Proceedings of the Royal Society of London B., 270, 313–321. 10.1098/rspb.2002.2218 PMC1691236 12614582

[ece36733-bib-0036] Hewitt, G. M. (1996). Some genetic consequences of ice ages, and their role in divergence a speciation. Biological Journal of the Linnean Society, 58, 247–276.

[ece36733-bib-0037] Hewitt, G. M. (1999). Post‐glacial re‐colonization of European biota. Biological Journal of the Linnean Society, 68, 87–112. 10.1111/j.1095-8312.1999.tb01160.x

[ece36733-bib-0038] Huemer, P. , Mutanen, M. , Sefc, K. M. , & Hebert, P. D. N. (2014). Testing DNA barcode performance in 1000 species of European Lepidoptera: Large Geographic distances have small genetic impacts. PLoS One, 9, e115774 10.1371/journal.pone.0115774 25541991 PMC4277373

[ece36733-bib-0039] Jinbo, U. , Kato, T. , & Ito, M. (2011). Current progress in DNA barcoding and future implications for entomology. Entomological Science, 14, 107–124. 10.1111/j.1479-8298.2011.00449.x

[ece36733-bib-0040] Jones, M. , Ghoorah, A. , & Blaxter, M. (2011). jMotu and Taxogenerator: Turning DNA barcode sequences into annotated operational taxonomic units. PLoS One, 6, e19259. 21541350 10.1371/journal.pone.0019259 PMC3081837

[ece36733-bib-0041] Kimura, M. (1980). A simple method for estimating evolutionary rates of base substitutions through comparative studies of nucleotide. Journal of Molecular Evolution, 16, 111–120. 7463489 10.1007/BF01731581

[ece36733-bib-0042] Koster, E. A. (2005). The physical geography of Western Europe. Oxford, UK: Oxford Regional Environments.

[ece36733-bib-0043] Kumar, S. , Strecher, G. , & Tamura, K. (2016). MEGA7: Molecular Evolutionary Genetics Analysis version 7.0 for bigger datasets. Molecular Biology and Evolution, 33, 1870–1874. 10.1093/molbev/msw054 27004904 PMC8210823

[ece36733-bib-0044] Lanfear, R. , Calcott, B. , Ho, S. Y. , & Guindon, S. (2012). PartitionFinder: Combined selection of partitioning schemes and substitution models for phylogenetic analyses. Molecular Biology and Evolution, 29, 1695–1701. 10.1093/molbev/mss020 22319168

[ece36733-bib-0045] Lemos, L. N. , Fulthorpe, R. R. , Triplett, E. W. , & Roesch, L. F. W. (2011). Rethinking microbial diversity analysis in the high throughput sequencing era. Journal of Microbiological Methods, 86, 42–51. 10.1016/j.mimet.2011.03.014 21457733

[ece36733-bib-0046] Leys, M. , Keller, I. , Räsänen, K. , Gattolliat, J. L. , & Robinson, C. T. (2016). Distribution and population genetic variation of cryptic species of the Alpine mayfly *Baetis alpinus* (Ephemeroptera: Baetidae) in the Central Alps. BMC Evolutionary Biology, 16, 77 10.1186/s12862-016-0643-y 27068234 PMC4828801

[ece36733-bib-0047] Lukhtanov, V. A. , Sourakov, V. , Zakharov, E. V. , & Herbert, P. D. N. (2009). DNA barcoding Central Asian butterflies: Increasing geographical dimension does not significantly reduce the success of species identification. Molecular Ecology Resources, 9, 1302–1310. 10.1111/j.1755-0998.2009.02577.x 21564901

[ece36733-bib-0048] Maddison, D. R. , & Maddison, W. P. (2005). MacClade 4: Analysis of phylogeny and character evolution. Version 4.08a. Retrieved from http://macclade.org 10.1159/000156416 2606395

[ece36733-bib-0049] Magri, D. , Fineschi, S. , Bellarosa, R. , Buonamici, A. , Sebastiani, F. , Schirone, B. , … Vendramin, G. G. (2007). The distribution of *Quercus suber* chloroplast haplotypes matches the palaeogeographical history of the western Mediterranean. Molecular Ecology, 16, 5259–5266. 17995923 10.1111/j.1365-294X.2007.03587.x

[ece36733-bib-0050] Meyer, C. , & Paulay, G. (2005). DNA barcoding: Error rates based on comprehensive sampling. PLOS Biology, 3, e422 10.1371/journal.pbio.0030422 16336051 PMC1287506

[ece36733-bib-0051] Monaghan, M. T. , Wild, R. , Elliot, M. , Fujisawa, T. , Balke, M. , Inward, D. J. G. , … Vogler, A. P. (2009). Accelerated species inventory on Madagascar using coalescent‐based models of species delineation. Systematic Biology, 58, 298–311. 10.1093/sysbio/syp027 20525585

[ece36733-bib-0052] Murienne, J. , & Giribet, G. (2009). The Iberian Peninsula: Ancient history of a hot spot of mite harvestmen (Arachnida: Opiliones: Cyphophthalmi: Sironidae) diversity. Zoological Journal of the Linnean Society, 156, 785–800. 10.1111/j.1096-3642.2008.00512.x

[ece36733-bib-0053] Nicholls, J. A. , Challis, R. J. , Mutun, S. , & Stone, G. N. (2012). Mitochondrial barcodes are diagnostic of shared refugia but not species in hybridizing oak gall wasps. Molecular Ecology, 21, 4051–4062. 10.1111/j.1365-294X.2012.05683.x 22724511

[ece36733-bib-0054] Paradis, E. , Claude, J. , & Strimmer, K. (2004). APE: Analyses of phylogenetics and evolution in R language. Bioinformatics, 20, 289–290. 10.1093/bioinformatics/btg412 14734327

[ece36733-bib-0055] Pentinsaari, M. , Hebert, P. D. N. , & Mutanen, M. (2014). Barcoding Beetles: A Regional Survey of 1872 Species Reveals High Identification Success and Unusually Deep Interspecific Divergences. PLoS One, 9, e108651 10.1371/journal.pone.0108651 25255319 PMC4177932

[ece36733-bib-0056] Pinheiro, J. , Bates, D. , De Roy, S. , Sarkar, D. , & R Core Team (2017). nlme: Linear and nonlinear mixed effects models. R package version, 3, 1–131. Retrieved from https://CRAN.R‐project.org/package=nlme

[ece36733-bib-0057] Pinto, M. A. , Muñoz, I. , Chávez‐Galarza, J. , & De la Rúa, P. (2012). The Atlantic side of the Iberian Peninsula: A hot‐spot of novel African honey bee maternal diversity. Apidologie, 43, 663–673. 10.1007/s13592-012-0141-1

[ece36733-bib-0058] Pons, J. , Barraclough, T. G. , Gomez‐Zurita, J. , Cardoso, A. , Duran, D. P. , Hazell, S. , … Vogler, A. P. (2006). Sequence‐based species delimitation for the DNA taxonomy of undescribed insects. Systematic Biology, 55, 595–609. 10.1080/10635150600852011 16967577

[ece36733-bib-0059] Puillandre, N. , Lambert, A. , Brouillet, S. , & Achaz, G. (2016). ABGD, Automatic Barcode Gap Discovery for primary species delimitation. Molecular Ecology, 21, 1864–1877. 10.1111/j.1365-294X.2011.05239.x 21883587

[ece36733-bib-0060] QGIS Development Team (2009). QGIS Geographic Information System. *OsGeo* Retrieved from http://qgis.osgeo.org/

[ece36733-bib-0061] R Core Team (2016). R: A language and environment for statistical computing. Vienna, Austria: R Foundation for Statistical Computing Retrieved from http://www.r‐project.org

[ece36733-bib-0062] Rambaut, A. , Suchard, M. A. , Xie, D. , & Drummond, A. J. (2014). Tracer v1.6. Retrieved from http://tree.bio.ed.ac.uk/software/tracer/

[ece36733-bib-0063] Ratnasingham, S. , & Hebert, P. D. N. (2007). BOLD: The barcode of life data system. Molecular Ecology Notes, 7, 355–364. Retrieved from www.barcodinglife.org 18784790 10.1111/j.1471-8286.2007.01678.x PMC1890991

[ece36733-bib-0064] Ratnasingham, S. , & Herbert, P. D. N. (2013). A DNA‐based registry for all animal species: The Barcode Index Number (BIN). PLoS One, 8, e66213. 23861743 10.1371/journal.pone.0066213 PMC3704603

[ece36733-bib-0065] Robusto, C. (1957). The cosine‐haversine formula. The American Mathematical Monthly, 64, 38–40. 10.2307/2309088

[ece36733-bib-0066] Ruiz‐Carbayo, H. , Bonal, R. , Espelta, J. M. , Hernández, M. , & Pino, J. (2017). Community assembly in time and space: The case of Lepidoptera in a *Quercus ilex* L. savannah‐like landscape. Insect Conservation and Diversity, 10, 21–31.

[ece36733-bib-0067] Savolainen, V. , Cowan, R. S. , Volger, A. P. , Roderick, G. K. , & Lane, R. (2005). Towards writing the encyclopedia of life: An introduction to DNA barcoding. Philosophical Transactions of the Royal Society B: Biological Sciences, 360, 1805–1811. 10.1098/rstb.2005.1730 PMC1609222 16214739

[ece36733-bib-0068] Schmitt, T. (2007). Molecular biogeography of Europe: Pleistocene cycles and postglacial trends. Frontiers in Zoology, 4, 11 10.1186/1742-9994-4-11 17439649 PMC1868914

[ece36733-bib-0069] Schroeder, H. , & Degen, B. (2008). Spatial genetic structure in populations of the green oak leaf roller, *Tortrix viridana* L. (Lepidoptera, Tortricidae). European Journal of Forest Research, 127, 447–453. 10.1007/s10342-008-0228-4

[ece36733-bib-0070] Shen, Y. , Guan, L. , Wang, D. , & Gan, X. (2016). DNA barcoding and evaluation of genetic diversity in Cyprinidae fish in the midstream of the Yangtze River. Ecology Evolution, 9, 2702–2713. 10.1002/ece3.2060 PMC4798831 27066250

[ece36733-bib-0071] Sihvonen, P. , & Skou, P. (2015). The geometrid Moths of Europe: Volume 5. Subfamily Ennominae I. Leiden, the Netherlands: Brill.

[ece36733-bib-0072] Soria, S. (1988). Lepidópteros defoliadores de Quercus pyrenaica, Willdenow 1805. Madrid, Spain: Ministerio de Agricultura, Pesca y Alimentación, Dirección General de la Producción Agraria, Subdirección General de Sanidad Vegetal.

[ece36733-bib-0073] Spouge, J. L. (2016). Measurement of a Barcode’s accuracy in identifying species In Trivedi S. et al (Eds.), DNA barcoding in marine perspectives (pp. 29–41). Cham, Switzerland: Springer International Publishing.

[ece36733-bib-0074] Stahlhut, J. K. , Fernández‐Triana, J. , Adamowicz, S. J. , Buck, M. , Goulet, H. , Hebert, P. D. , … Smith, M. A. (2013). DNA barcoding reveals diversity of Hymenoptera and the dominance of parasitoids in a sub‐arctic environment. BMC Ecology, 13, 2. 23351160 10.1186/1472-6785-13-2 PMC3565895

[ece36733-bib-0075] StatSoft, Inc. (2005). STATISTICA (data analysis software system), version 7.1. Retrieved from www.statsoft.com

[ece36733-bib-0076] Taberlet, P. , Fumagalli, L. , Wurst‐Saucy, A. G. , & Cosson, J. F. (1998). Comparative phylogeography and postglacial colonization routes in Europe. Molecular Ecology, 7, 453–464. 10.1046/j.1365-294x.1998.00289.x 9628000

[ece36733-bib-0077] Talavera, G. , Dincă, V. , & Vila, R. (2013). Factors affecting species delimitations with the GMYC model Insights from a butterfly survey. Methods in Ecology and Evolution, 4, 1101–1110. 10.1111/2041-210X.12107

[ece36733-bib-0078] Tang, C. Q. , Obertegger, U. , Fontaneto, D. , & Barraclough, T. G. (2014). Sexual species are separated by larger genetic gaps than asexual species in rotifers. Evolution, 68, 2901–2916. 10.1111/evo.12483 24975991 PMC4262011

[ece36733-bib-0079] Thompson, J. N. , & Pellmyr, O. (1991). Evolution of oviposition behavior and host preference in Lepidoptera. Annual Review of Entomology, 36, 65–89. 10.1146/annurev.en.36.010191.000433

[ece36733-bib-0080] Torres‐Vila, L. M. , & Bonal, R. (2019). DNA barcoding of large oak‐living cerambycids: Diagnostic tool, phylogenetic insights and natural hybridization between *Crambyx cerdo* and *Cerambyx welensii* (Coleoptera: Cerambycidae). Bulletin of Entomological Research, 109, 583–594. 10.1017/S0007485318000925 30514408

[ece36733-bib-0081] Villesen, P. (2007). FaBox: An online fasta sequence toolbox. Retrieved from http://www.birc.au.dk/software/fabox

[ece36733-bib-0082] Wright, S. (1943). Isolation by distance. Genetics, 3, 114–138. 10.1093/genetics/28.2.114 PMC1209196 17247074

