# Supplementary material for: DNA Barcoding and geographical scale effect: The problems of undersampling genetic diversity hotspots
Source: Ecol Evol. 2020 Sep 1;10(19):10754–72. doi: 10.1002/ece3.6733 (PMC7548170; doi:10.1002/ece3.6733)
Supplement: Supplementary file 1 — Supplementary Material [file ECE3-10-10754-s001.docx]

**DNA Barcoding and geographical scale effect: the problems of undersampling genetic diversity hotspots**

**Á. Gaytán, J. Bergsten, T. Canelo, C. Pérez-Izquierdo, M. Santoro, R. Bonal**

**APPENDIX S1: BOLD process IDs**

ABOLA923-15, ABOLA924-15, BTM001-10, BTM011-10, BTM014-10, BTM023-10, BTM026-10, BTM027-10, BTM032-10, BTM039-10, BTM040-10, BTM048-10, BTM049-10, BTM050-10, BTM051-10, BTM052-10, BTM053-10, BTM054-10, BTM055-10, BTM056-10, BTM057-10, BTM058-10, BTM076-10, BTM077-10, BTM078-10, BTM079-10, BTM080-10, BTM081-10, BTM082-10, BTM083-11, BTM084-10, BTM085-10, BTM092-10, CGUKA141-09, CGUKA293-09, CGUKA326-09, CGUKA329-09, CGUKA346-09, CGUKA358-09, CGUKA468-09, CGUKA498-09, CGUKA528-09, CGUKA885-09, CGUKAB153-09, CGUKB346-09, CGUKC021-09, CGUKC031-09, CGUKC1024-09, CGUKC1043-09, CGUKC110-09, CGUKC279-09, CGUKC414-09, CGUKC451-09, CGUKC474-09, CGUKC570-09, CGUKC732-09, CGUKC968-09, CGUKD482-09, CGUKD497-09, CGUKD575-09, CGUKD812-09, CGUKD824-09, EII361-15, EII387-15, EII396-15, EII398-15, EII427-15, EII429-15, EII433-15, EII466-15, EII529-15, EII536-15, EII567-15, EII601-15, EII610-15, EII616-15, EII655-15, EII682-15, EII707-15, EII722-15, EII729-15, EII732-15, EII734-15, EII741-15, EII745-15, EII752-15, FBLMS097-09, FBLMS303-09, FBLMT137-09, FBLMT581-09, FBLMU056-09, FBLMU524-09, FBLMU945-09, FBLMV208-09, FBLMV750-09, FBLMV752-09, FBLMZ269-12, FBLMZ462-12, FBLMZ476-12, GBLAA227-14, GBLAA366-14, GBLAA448-14, GBLAA989-14, GBLAA990-14, GBLAB035-13, GBLAB587-13, GBLAB617-13, GBLAC011-13, GBLAC012-13, GBLAC013-13, GBLAC062-13, GBLAC1087-13, GBLAC247-13, GBLAC358-13, GBLAC362-13, GBLAC835-13, GBLAC963-13, GBLAD136-14, GBLAD137-14, GBLAF256-14, GBLAF285-14, GBLAF421-14, GBLAF429-14, GBLAF587-14, GBLAF595-14, GBLGC217-12, GBLGC264-12, GMGMK811-14, GWOAL066-08, GWOAL067-08, GWOR069-07, GWOR070-07, GWOR071-07, GWOR072-07, GWOR073-07, GWOR074-07, GWOR4078-09, GWOR4168-09, GWOR4215-09, GWORA2528-09, GWORA2985-15, GWORA650-08, GWORA999-08, GWORB035-07, GWORB1560-08, GWORB1561-08, GWORB1562-08, GWORC001-07, GWORD2059-08, GWORD640-08, GWORD641-08, GWORD752-08, GWORK346-09, GWORM221-09, GWORM278-09, GWORM816-09, GWORP983-09, GWORR862-10, GWORR863-10, GWORU351-10, GWORU379-10, GWORZ097-10, GWORZ149-10, GWORZ489-10, GWOSA415-10, GWOSI022-10, GWOSP890-11, GWOSP929-11, GWOTF286-12, GWOTF300-12, GWOTF324-12, GWOTH922-12, GWOTH923-12, GWOTH930-12, GWOTL137-13, GWOTL187-13, GWOTL276-13, GWROB1831-08, IBLAO564-12, LASTS431-14, LASTS637-14, LASTS638-14, LEATA002-13, LEATA003-13, LEATA036-13, LEATA038-13, LEATA083-13, LEATB913-13, LEATE167-13, LEATF374-14, LEATF381-14, LEATF423-14, LEATG558-14, LEATH447-14, LEATH448-14, LECRT090-15, LEFIA1403-10, LEFIA880-10, LEFIA890-10, LEFIB256-10, LEFIB787-10, LEFIC659-10, LEFIC660-10, LEFIC661-10, LEFIC811-10, LEFIC815-10, LEFID681-10, LEFIE585-10, LEFIF539-10, LEFIH005-10, LEFIH006-10, LEFIJ586-10, LEFIJ592-10, LEFIJ593-10, LEFIK932-10, LEFIK944-10, LENOA1078-11, LENOA1079-11, LENOA286-11, LENOA287-11, LENOA288-11, LENOA289-11, LENOA290-11, LENOA377-11, LENOA467-11, LENOA649-11, LENOA671-11, NLLEA052-12, NLLEA058-12, NLLEA059-12, NLLEA060-12, NLLEA068-12, NLLEA112-12, NLLEA113-12, NLLEA1138-12, NLLEA1185-12, NLLEA383-12, NLLEA639-12, NLLEA643-12, NLLEA650-12, NLLEA652-12, NLLEA654-12, NLLEA658-12, NLLEA679-12, NLLEA680-12, NLLEA681-12, NLLEA704-12, NLLEA782-12, NLLEA797-12, NOCJH375-10, NOCJH384-11, NOCJH385-11, ODOPE319-11, ODOPE730-11, PHLAA405-09, PHLAA473-09, PHLAB1219-10, PHLAC090-10, PHLAC119-10, PHLAC123-10, PHLAC482-10, PHLAC606-10, PHLAE006-11, PHLAE007-11, PHLAE022-11, PHLAE474-11, PHLAF192-11, PHLAF260-11, PHLAI313-13, PHLAV194-12, PHLAV200-12, PHLAV286-12, PHLSA538-11, PHLSA570-11, PHLSA572-11, PHLSA637-11, PHLSA667-11.
